# A Smart IoT System for Detecting the Position of a Lying Person Using a Novel Textile Pressure Sensor

**DOI:** 10.3390/s21010206

**Published:** 2020-12-31

**Authors:** Robert Hudec, Slavomír Matúška, Patrik Kamencay, Miroslav Benco

**Affiliations:** Faculty of Electrical Engineering and Information Technology, University of Zilina, 01026 Zilina, Slovakia; robert.hudec@uniza.sk (R.H.); slavomir.matuska@uniza.sk (S.M.); miroslav.benco@uniza.sk (M.B.)

**Keywords:** smart sensor, IoT system, Velostat, pressure sensor, convolutional neural network, data classification, position detection

## Abstract

Bedsores are one of the severe problems which could affect a long-term lying subject in the hospitals or the hospice. To prevent lying bedsores, we present a smart Internet of Things (IoT) system for detecting the position of a lying person using novel textile pressure sensors. To build such a system, it is necessary to use different technologies and techniques. We used sixty-four of our novel textile pressure sensors based on electrically conductive yarn and the Velostat to collect the information about the pressure distribution of the lying person. Using Message Queuing Telemetry Transport (MQTT) protocol and Arduino-based hardware, we send measured data to the server. On the server side, there is a Node-RED application responsible for data collection, evaluation, and provisioning. We are using a neural network to classify the subject lying posture on the separate device because of the computation complexity. We created the challenging dataset from the observation of twenty-one people in four lying positions. We achieved a best classification precision of 92% for fourth class (right side posture type). On the other hand, the best recall (91%) for first class (supine posture type) was obtained. The best F1 score (84%) was achieved for first class (supine posture type). After the classification, we send the information to the staff desktop application. The application reminds employees when it is necessary to change the lying position of individual subjects and thus prevent bedsores.

## 1. Introduction

The expected population development shows that the process of population aging will intensify in the coming years and will deepen long into the future. Therefore, one of the state authorities’ priorities is the aging of the population and the quality of their life. This priority is focusing on the health insurance of older fellow citizens, including assistance in the field of social security. A lot of elderly people rely on the help of either their family members or the hospice employees. Many of them are long-term lying subjects for health reasons. There is a significant risk of bedsores in long-term lying subjects. This risk is also present in patients hospitalized for a long time. The problems described above are the primary motivation for this article. To prevent lying bedsores, we have designed a smart IoT system for detecting the position of a lying person using novel textile pressure sensors. The proposed system is intended to help hospital and hospice staff, but also family members who take care of a person in the prevention of lying bedsores. Sixty-four novel textile pressure sensors placed on the mattress toppers provide us with information about the position of the lying subject. We transfer this information to the cloud, evaluate it, and then display it on a desktop application. The application reminds employees when it is necessary to change the lying position of individual subjects and thus prevent bedsores.

There are several papers published with a similar topic to our proposed complex solution. Authors in [1] presented a complex system based on an artificial neural network for in-bed posture classification. Unlike our solution, they used the commercially available pressure sensor mattress. The mattress consists of a 2.5 mm-thick bedsheet containing a matrix of 64 × 27 textile made piezo-resistive pressure sensors. They carried out the experiments on 12 healthy adults using histogram of gradients and local binary patterns feature extraction and feed-forward artificial neural network as a classification method. The method classifies the lying posture into one of the four postures (supine, prone, left, and right lateral side). They achieved accuracy at 97.9%. As the authors stated in the conclusion, their presented work did not offer a comprehensive solution to the problem. It presents a potential technique to make a fundamental step towards prevention.

Authors in [2,3,4] proposed the solution based on their own version of the bedsheet-sensing element. Hong [2], in his research article, presents a smart care bed for elderly patients. The smart bed features several pressure sensors FSR 408 and FSR 406. Their primary motivation was to prevent falls and bedsores. As the final implementation, he presented a real-time pressure-sensing algorithm capable of deciding on the possibilities of bedsores and falling accidents by considering both the intensity and the duration of the pressure of specific body parts. Abdelmoghith et al. [3] provide a solution that reduces the possibility of developing bedsores for long-term patients. They created an IoT-based healthcare monitoring system based on their prototype of a mattress. They used FSR 406 pressure sensors for body pressure distribution and DHT11 for temperature measurement. The proposed system did not classify the lying person’s posture but only monitored the subject’s mobility. The system provides crucial information using a mobile application based on the collected data. Authors in [4] also use the sensors from the FSR family to determine the pressure distribution of the lying person. They demonstrated the concept of a pressure sensing/monitoring system for pressure ulcer prevention. They equipped the bedsheet with 16 sensors and measured their resistance using an Arduino-based board. However, they did not solve the problem of laying person posture classification and provide only the concept of smart bedsheet.

The findings presented in [5] offer another approach to preventing bedsores. They used wearable computing and a deep learning approach to pressure ulcer prevention. To get the information about the lying person, they used a non-invasive system of wearable sensors based on inertial sensor. They can estimate the positions of the patients, and send an alert signal when the subject remains in the same position for too long a period. The disadvantage of this solution is that the lying person has to wear the same piece of electronic equipment. Authors in [6] propose a similar solution. They used a comprehensive approach for designing a sensor system that uses a single accelerometer along with machine learning algorithms for in-bed lying posture classification. They evaluated nine different accelerometer positions on the human body and they achieved the best F1 scores at 97.8% in lying postures detection.

In the papers mentioned above, authors use commercially available pressure sensors in their prototypes or accelerometer to get information about the lying person. On the other hand, the works presented in [7,8,9] propose the solution for pressure distribution measurement based on their own sensors. For instance, Saenz-Cogollo et al. [7] present a pressure mapping mat for tele-home care applications. They developed a mat-like pressure mapping system based on a single layer textile sensor. The sensor is fabricated by embroidering silver-coated yarns on a piece of light cotton fabric and creating pressure-sensitive resistive elements by stamping the conductive polymer at the crossing points of conductive stitches. The mat consists of an array of 32 × 32 sensing elements. They demonstrated the functionality and performance by comparing the proposed mat with the commercial pressure platform. Li et al. [8] present a low-cost textile smart map for step pressure sensing based on multiple layer structure with polyester textile, conductive copper taffeta, and Velostat. The matrix of sensors consists of 8 × 10 sensing points. They used an Arduino-based board for transferring the measured data into the personal computer for evaluation and visualization. The study presented in [9] describes a low-cost smart portable sitting mat that can measure the sitting plantar pressure distribution in real-time. The smart mat consists of 576 pressure-sensing elements made by a low-cost E-Textile. The presented solution uses voltage feedback non-scanning electrode to eliminate the cross-talk effect between the sensing elements. The primary goal of the presented work is the creation of a low-cost and effective sensing pressure mat.

Our system relies on novel textile pressure sensors based on electrically conductive yarn and the Velostat. Suprapto et al., in their study [10], also rely on the Velostat-based pressure sensors. They presented a characteristic of a 32 × 32 sensor matrix for foot pressure distribution measurements. The single proposed sensor is constructed from dual electrodes placed on the top and underneath Velostat as the sensing material with a size of 7 × 7 mm. Based on their findings, the proposed sensor shows a relatively linear force–conductance response. A similar sensor design is presented by the authors in [11]. Their sensor is an easy-to-build textile pressure sensor created from low-cost conventional anti-static sheets and conductive woven fabrics. The sensor consists of three layers, the top conductive layer, the middle low-density polyethylene with a carbon sheet, and the bottom conductive layer. The size of the sensor is approximately 23 × 23 mm. From the evaluated characteristics, they concluded that the proposed design is suitable for didactic, healthcare, and lifestyle applications. Authors in [12,13] also described Velostat-based pressure sensors; moreover, the sensor design pattern is the same. The sensor consists of three layers, where the Velostat is in the middle. Tihak and Boskovic [14] carried out the experiments demonstrating the development of pressure sensor using Velostat. They presented typical problems and also discussed the causes and possible remedies. Lin and Seet [15] present an improved design of a textile pressure sensor. The sensor is constructed of conductive yarns and a dual-layer piezoresistive polymer (Velostat) as the sensing material. The sensor size is 10 × 10 mm. The proposed sensor can detect the load up to 1000 kPa with a relatively linear response.

The structure of our paper is as follows. The next chapter describes the IoT system proposal in detail, the novel textile pressure sensors development, hardware development, and whole topper production. Software descriptions follow next. We will give a view of how communications work between the system parts and will describe the Node-RED and desktop applications. Then, we will present the neural network used for lying posture classification and achieved results from our experiments. The discussion and conclusion section are at the end of our paper.

## 2. IoT System Proposal

**Figure 1:**
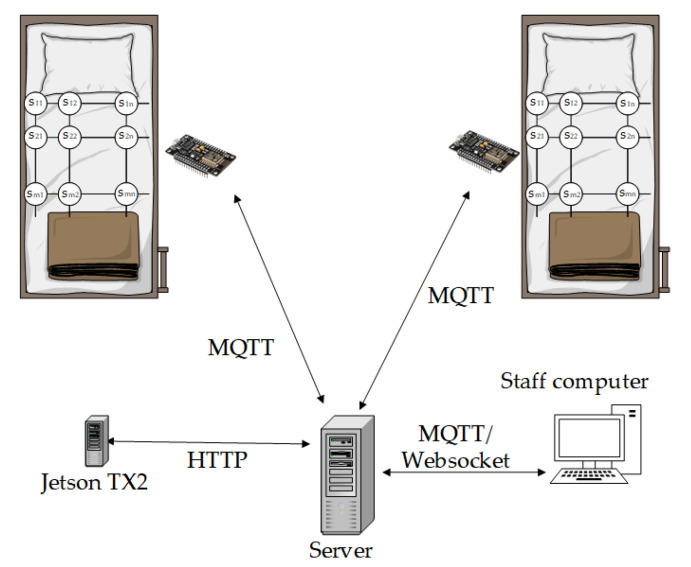
A smart IoT-based system concept proposal.

Our primary goal was to create a smart IoT-based system for the detection of the position of a laying person in bed. The system should help staff who take care of long-term lying persons. The main task is to warn the staff that the person is lying in one position for a long time and it is necessary to change the lying person’s position. In this way, it is possible to prevent unwanted bedsores. Figure 1 shows the proposal for the system concept.

The system consists of one or more Arduino-based hardware solutions with attached pressure sensors on the mattress topper, the server, computation development board, and software application on the personal computer (PC). For every bed, 64 novel sensors detect the pressure distribution. Because the bed is usually placed in the same place, the hardware solution runs from the electrical network and does not need a battery power source. The network-attached storage from QNAP holds the server solution. It features the Message Queuing Telemetry Transport (MQTT) broker for communication, Node-RED for the logic, and Mongo database for data storage. For the position classification, we use the neural network. The neural network needs a lot of computation power. Because of this requirement, computation runs on a separate device.

In our case, it is the Nvidia Jetson TX2 development board. On the PC runs a Qt-based desktop application where the user interacts with the system. The communication flow is as follows:

- Arduino collects and sends pressure distribution to the server.
- Server receives and stores the data. Then the server requests the development board for data evaluation.
- The user gets all information from the server in the desktop application on the PC.

We will provide detailed information about the individual parts of our solution in the next sections.

### 2.1. Sensor Design

**Figure 2:**
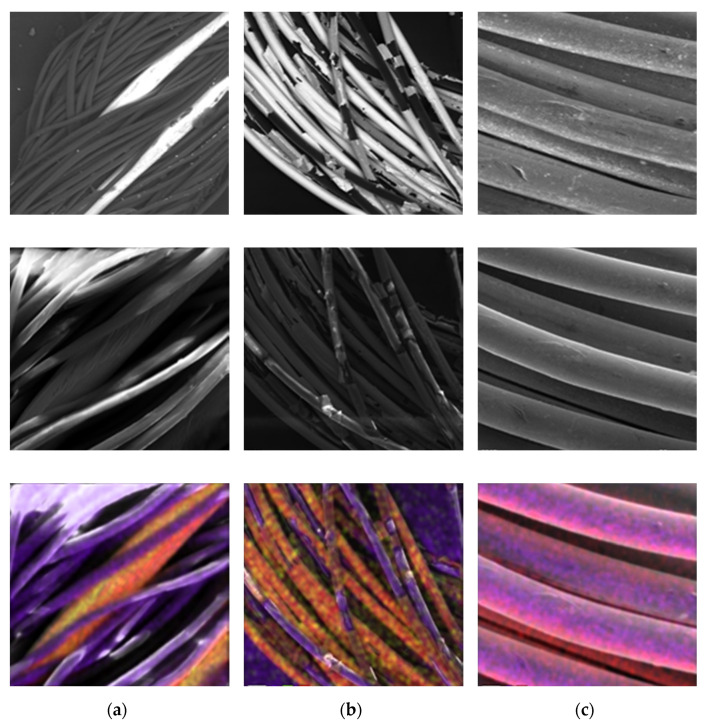
Electroconductive yarns with chemical analysis. (**a**) Two 50 mm Cu/Ag wires blended with polyester yarns; (**b**) Elitex yarn; (**c**) 3 ltex yarn with antioxidant treatment.

The overall design of an intelligent topper should meet several requirements that are critical to its conventional use. One of the main requirements for long-term use is the application of an electrically conductive thread, which is not significantly damaged by abrasion and common detergents. Other relevant parameters are simple industrial production, low consumption of electrically conductive textile materials, the comfort of a person lying on a bed, or resistance of the conductive paths of the mattress to mechanical damage or tearing. In our designs and experiments, we tested three electroconductive yarns. Figure 2a shows two 50 mm copper wires with a silver coating blended with polyester yarns. Figure 2b illustrates the Elitex yarn based on polyamide yarns by a chemical silver coating. Figure 2c shows the third yarn, 3 ltex yarn based on two blended multifilaments of polyamide yarns by chemical silver coating with antioxidant treatment.

Based on our experiences with sewing electroconductive patterns, we chose the 3 eltex yarn with antioxidant treatment. Its core consists of polyamide coated by pure silver with a fineness of 278 dtex and surface electrical resistance of 260 Ω/m. The yarns based on the two blended 50 µm Cu/Ag wire with polyester yarns are uncomfortable for humans. Similarly, the Elitex yarn is not suitable for long-time use. The reason is the peeling of the silver layer from the surface of the fiber during washing and subsequent mechanical stress. The selected yarn is gentle on the human skin, it is more resistant to the oxidation process, and it has better embroidery properties. Our motivation was to choose a yarn that will be suitable for sewing with embroidery machines, and the design pattern will be strong enough and reliable even in cases of an extreme load, and also resistant to ordinary.

**Figure 3:**
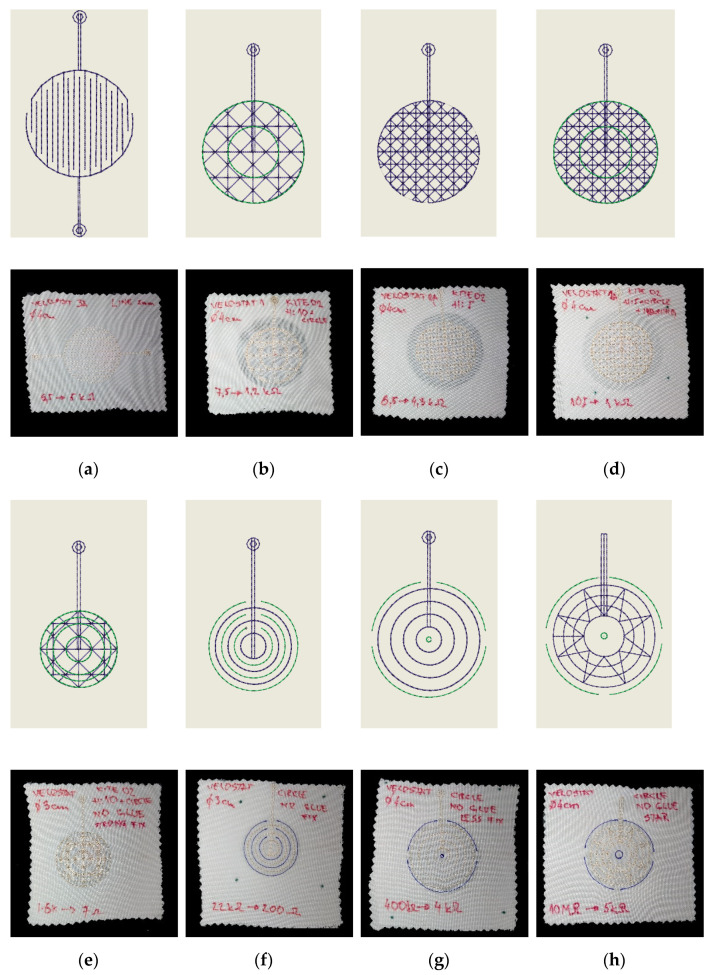
Sensing node designs in Wilcom DecoStudio and their fabrications. (**a**) PC2D; (**b**) PC3H; (**c**) PC3I; (**d**) PC3J; (**e**) PC3K; (**f**) PR4A; (**g**) PR4B; (**h**) PR4C.

The primary sensor design was based on the change of resistance or capacitance principle. Moreover, electrodes will be embroidered in one step, fully textile, and fabricated on one or two textile layers. Following these conditions, we have developed and fabricated more than 30 designs. We focused on determining the influence of various factors that affect the production process or the overall parameters of the sensor. These include, for example, the stitch type and the number of yarn cross-connections, the type, and construction of electroconductive yarn, embroidering speed, and continuity of seeding with minimizing the number of yarn cuts, time and temperature of ironing. Figure 3 shows relevant milestone designs. The sensor abbreviation is explained in the following Table 1.

The sensing place at the topper covers approximately 0.7 m^2^. This is an area where a person lies with an average figure and a weight of 90 kg. The sensing surface on which a person lies is defined by his/her shoulders and buttocks. Moreover, this part of a person’s body accounts for about 60 percent of a person’s total weight with an average load of approximately 7 g/cm^2^. In experiments, we used a load weight of 11.5 kg to simulate the pressure of the human body. The load is placed on the sensor through a dielectric plate with a size of 40 × 40 mm which reaches a pressure equivalent to 100 times the human load (718 g/cm^2^). It simulates extreme human strain and sensor resistance when a person is moving on a mattress. The goal is to achieve a change in measured values by at least two orders of magnitude with the lowest possible consumption of electrically conductive yarn. Table 2 presents the sensor’s details and measured values. We used Velostat as the middle layer between the top and bottom electrodes in the sensor design. Velostat is a piezo-resistive material with high volume resistivity (<500/cm) and a thickness of only 200 microns. The Velostat is pressure sensitive, so squeezing it will reduce the resistance.

**Figure 4:**
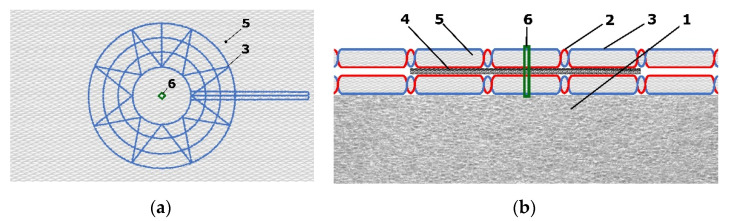
Measuring node realized by PR4C design. (**a**) Top view; (**b**) transversal view: (1) polyurethane foam, (2) electrically conductive yarn, (3) nonconductive yarn, (4) Velostat foil, (5) base fabric, (6) fixation with nonconductive yarn.

As it can be seen in Figure 4a, the design marked as PR4C achieved the highest change in resistance. Figure 4b illustrates the top and transversal view of the best design.

The developed primary sensor or sensing node of the intelligent topper should have the simplest design possible because it is a challenge to embroider a big sensor area. It will be used as a part of a sensing matrix of eight-by-eight sensing nodes, which should be sufficient to classify the person’s sleep posture and to detect his/her activity during the night.

### 2.2. Hardware Design

**Figure 5:**
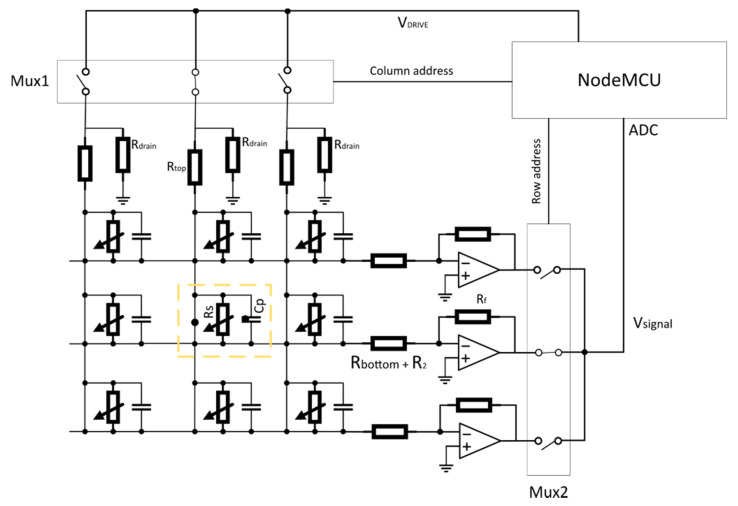
Schematic of the hardware solution based on multiplexers, the trans-impedance amplifiers, and NodeMCU board.

We described the pressure sensor in previous part. Now it is necessary to design hardware solutions to measure, collect, and send the data to the server. In our design, we used a NodeMcu microcontroller. The NodeMcu is an Arduino-based board where the central processor is ESP8266. The communication via Wi-Fi with the server is also provided by this processor. The main advantage of this board is that it can be directly connected to the Wi-Fi and processing the data from sensors at the same time from one source code. The board features 16 general-purpose input–output pins and one analog input. In the hardware design, we followed the findings presented in [10,11,12]. We developed an electronic setup, which can measure each sensor pressure in the matrix of sensors using multiplexers. Figure 5 shows the proposed schematic.

Each sensor in the matrix of sensors is represented by its resistance R_s_ in parallel with a parasitic capacitance C_p_. R_top_ and R_bottom_ represent the yarn resistance. While our proposed novel sensor decreases the resistance with the applied pressure, we adjust the sensor sensing ability by adding R_2_ resistance in the front of trans-impedance amplifiers. NodeMCU manages the Mux1 and Mux2 to select a particular sensor sequentially, where Mux1 is responsible for selecting the column in the matrix and Mux2 for the row. We use the resistance R_drain_ to reduce the effect of C_p_ by referring the non-active columns to the ground. To minimalize the cross-talk between rows of the matrix, we added the trans-impedance amplifiers for each row. Usage of the trans-impedance amplifiers allows us to measure voltage Vsignal that is inversely proportional to the measuring sensor resistance.

**Figure 6:**
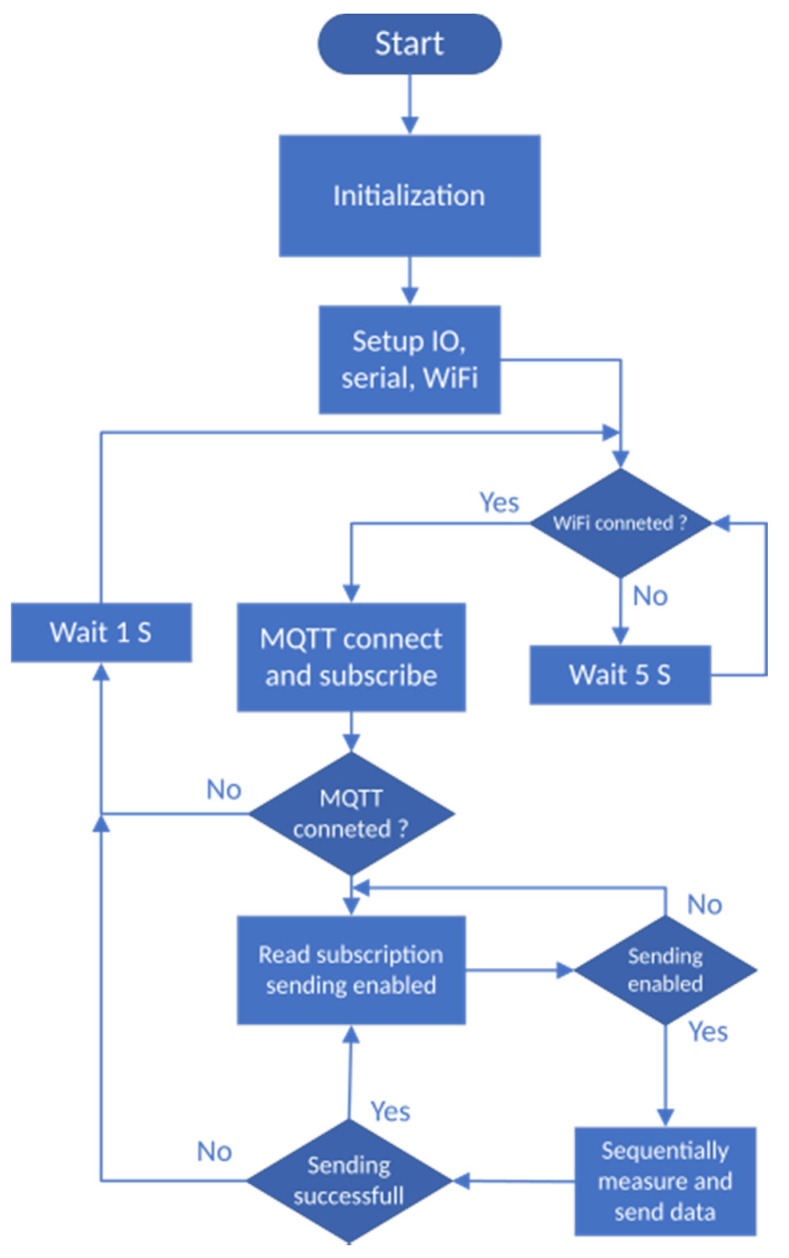
NodeMcu source code flowchart for pressure data acquisition and sending to the server.

The Figure 6 illustrates the source code flowchart diagram. The diagram starts with the initialization of all the necessary components. Then, the general input/output pins and communication peripheries enter the default state. Using provided Wi-Fi credentials, the NodeMcu connects to the Wi-Fi network. If the connection to the network fails, the system waits for 5 s and then repeats this operation until the successful login. The next step is a connection to the MQTT broker using pre-defined credentials. The MQTT protocol communication is provided by an external library—Adafruit MQTT Library ESP8266. To establish the MQTT connection, we need the client instance of the class Adafruit_MQTT_Client. The client connects to the MQTT broker. We have to create an additional object for receiving the responses from the broker. The instance of the class Adafruit_MQTT_Subscribe provides such an interface. An Adafruit_MQTT_Publish class provides the application programming interface (API) for sending data. After a successful connection, the object from Adafruit_MQTT_Subscribe class fetches the data from the MQTT broker using the channel identifier for reading commands. On the server side, the MQTT broker resends the commands from the desktop application on the PC. The valid commands are to start and stop the measurements. The measurement is performed in cycles every 1 s. We are using two analog multiplexers to select the particular sensor in the matrix of sensors on the output and input side.

The first multiplexer sets the output voltage on a specific output and similarly, the second multiplexer sets the specific input, which transfers the signal to the NodeMcu. On the NodeMcu, we are measuring the signal amplitude using an analog input pin. The signal amplitude increases with the dropping resistance. After finishing the measurement of the signals, NodeMcu initiates sequential data sending to the server. It is necessary to send data sequentially because of the processor ram limitation. The data are sent in JavaScript Object Notation (JSON) format using Adafruit_MQTT_Publish object.

### 2.3. Topper Design and Production

**Figure 7:**
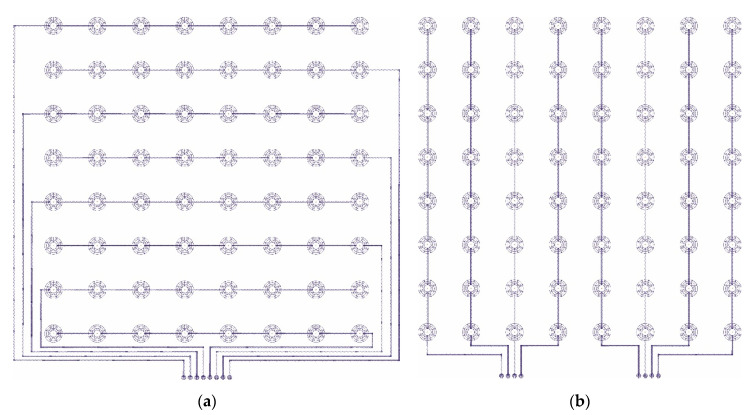
Final topper’s pressure matrix design with eight by eight sensing nodes. (**a**) Top layer; (**b**) bottom layer.

Based on realized experiments mentioned in Section 2.1, we decided to use the pressure sensor with Velostat foil. The overall topper’s design consists of a partial pressure sensor creating a structure of eight by eight sensing nodes. We set the sensor diameter to 40 mm with 100 mm distances in horizontal and vertical axes. Figure 7 shows the sensor design for both layers.

**Figure 8:**
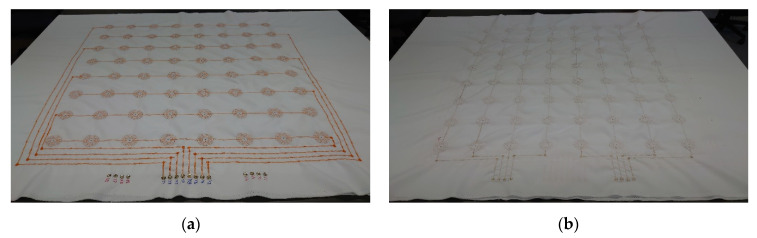
Embroidered patterns. (**a**) Top layer; (**b**) bottom layer.

The fabrication of the sensor starts by drawing a mesh of eight by eight dots on the base fabric. They mark the center of each sensing point. It is necessary because the overall sensor is too big to fabricate at one step and the material of the base fabric is a little bit elastic. Moreover, the precise final composition of both layers relative to one sensor at all 64 nodes is difficult to manufacture. We embroidered the partial sensors in the marked positions. In our prototype, we used the nonconductive viscose-based yarn for upper embroidery and silver-coated polyamide yarn as bottom embroidering filaments. After that, the rest of the connecting lines and connectors were embroidered too. It is a fact, that topper will be placed on the standard PolyURethane (PUR) foam mattress. From this point of view, the sensor prototype should be massive with a robust design able to resist a crackdown of the electroconductive yarns when a human is moving over the bed. For this reason, we secured the connections between sensor nodes by triple stitching. Figure 8 illustrates the fabrication of the sensor’s layers.

**Figure 9:**
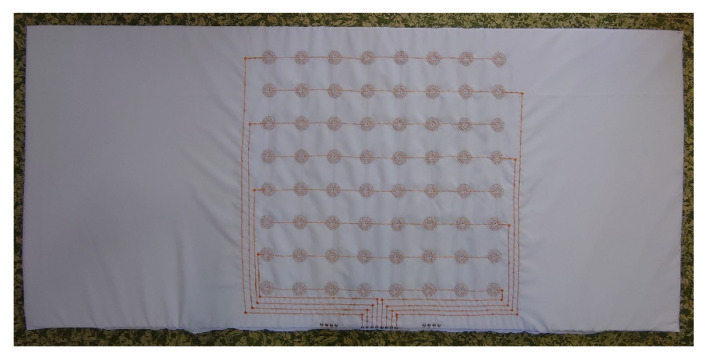
Final topper production applied on 40 mm polyurethane foam.

As the next step, we inserted circle Velostat foils between the fabricated layers in the positions of the sensors. Using the embroidery machine, we fixed both layers of the sensors. Finally, we stabilized the sensor matrix and applied it to the polyurethane foam. Figure 9 illustrates the final smart topper with our novel sensors.

The topper was created as a liner of polyurethane foam for a standard mattress of 2000 mm by 900 mm dimension. We chose to experimentally position it on the topper’s base fabric. The upper limit of the sensor location was determined by the shoulder position and its value was 550 mm from the edge of the topper. Likewise, the lower limit is determined by the buttocks at a distance of 1250 mm. The place between the shoulders and buttocks represents the dominant body footprint of a person.

Overall topper design was composed by Wilcom DecoStudio software, that produces a digital embroidery pattern of stitches. Both patterns have been stored on a PC hard drive as a file with EMB (Embroidery format) extension and DST (Data Stitch Tajima) for a programmable embroidery machine. Further, they were embroidered by Barudan BEXT-S1501CII separately.

### 2.4. IoT System Design

The central unit of our IoT system is the NAS from QNAP [16,17]. This unit runs programs and server services that provide connectivity, management, data storage, and communication between the system peripheries. Two primary services are running on the server:

- QIoT suite.
- Mongo DB [18,19].

**Figure 10:**
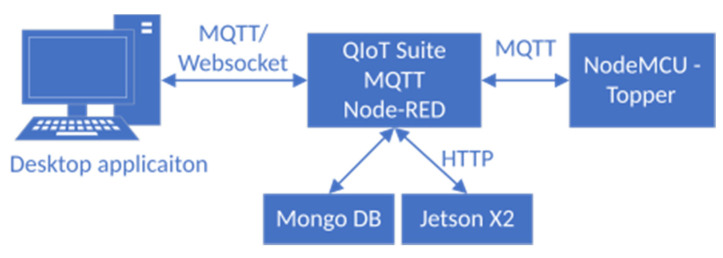
The system communication data flow between system components.

The QIoT suite is an application, which could be installed directly from the application center on NAS. QIoT suite is a complete and practical IoT private cloud platform for building and managing IoT applications. It integrates different services, which are necessary to provide a complex solution in the IoT world, into one application. The QIoT suite leverages popular tools like the MQTT broker [17,20], Node-RED, Freeboard, and supports multiple protocols and dashboards. MongoDB is a popular, general-purpose, document-based, distributed database, which is common in a cloud solution and IoT world. We use MongoDB for data storage and provision. Figure 10 shows the flowchart of the system data flow.

The primary data flow is as follows:

1. NodeMCU sends the measured data from the sensors to the QIoT using the MQTT protocol. On the QIot side, we are using Node-RED for data processing.
2. QIot receives the data, stores them to MongoDB, and issues the HTTP query to the Jetson X2 for data classification.
3. After the classification, QIot updates the record in the database with classified posture and sends the data to the desktop application.
4. The staff gets the current lying posture and other information.

#### 2.4.1. Node-RED Application

The Node-RED application implements the logic part of the IoT system for detecting the position of a lying person using the private cloud platform. In our solution, we used two primary flows with the deployed application:

- A data processing flow.
- A command processing flow.

**Figure 11:**
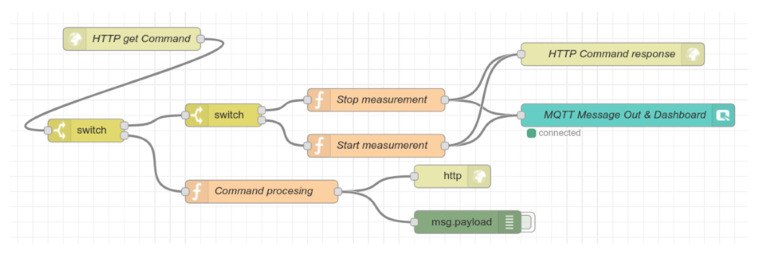
The Node-RED command processing flow for things based on HTTP request–response communication model.

Figure 11 shows the command processing flow. HTTP request–response communication model implements the command processing in our solution. The flow starts with the input HTTP node. There is one compulsory argument in the HTTP request—command type.

There are two valid values for command type, controlSending, and manageStorage. Use controlSending type to start or stop sending data from the topper. In this scenario, function Start measurement and Stop measurement prepares the command message and Qbroker out node sends the message to the topper. The arguments from the HTTP request appear in Node-RED as a JSON object. An example of such a JSON command is:

{
“type”:”controlSending”,
“cmd”:”1”
}

**Figure 12:**
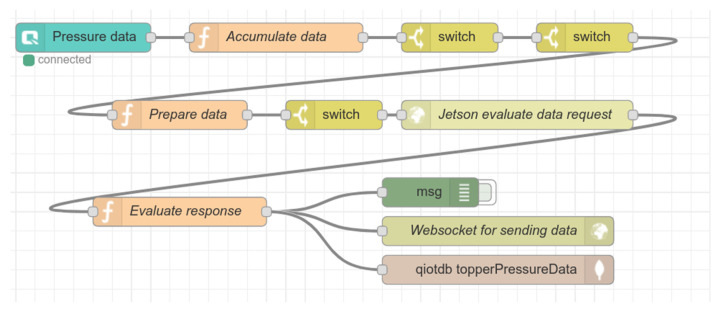
The Node-RED data processing flow for data collection, evaluation, propagation, and storage.

The manageStorage type serves as an API point during the experimental measurements and data acquisition for training and testing neural networks. We will describe this command type later in the section Data Acquisition. The Figure 12 illustrates the data processing flow.

The flow starts with Qbroker in node. This input node listens on the topic “pressureData.” The purpose of this flow is to collect, evaluate, and propagate the topper pressure data. The example of receiving data on the node is:

“payload”: {
“row”:4,
“data”: [“52.00”,”52.00”,”86.00”,”122.00”,”132.00”,”113.00”,”32.00”,”38.00”]
}

Because of the NodeMCU ram limitation, we have to send the measured data in sequences. Therefore we need eight messages to send data from one measurement. One message contains the pressure data from one row in the matrix of sensors. The row tag indicates the row position. The function Accumulate data serves as a buffer function for one measurement. The IP network could cause different delays and therefore function task is to check the row order, sort the data if necessary, and prepare data from one measurement for later evaluation. The next two switch blocks inspect the data format and integrity. The next block is the Prepare data function. The function checks the topper state and prepares the data for the evaluation in the neural network. Afterward, the switch block validates the topper state. HTTP request–response block sends the data for evaluation in JSON format as a parameter in the HTTP request to the Jetson module. Data looks like:

payload: {
timestamp: 1605683032,
subjectID: “subjet12”,
notes: ““,
data: [
[7, 8, 12, 12, 20, 123, 0, 0],
[8, 10, 27, 21, 28, 26, 0, 0],
[2, 9, 6, 8, 12, 8, 0, 0],
[6, 11, 11, 16, 22, 13, 3, 0],
[19, 32, 39, 95, 103, 66, 8, 0],
[13, 16, 16, 28, 51, 24, 1, 0],
[18, 20, 24, 47, 82, 40, 6, 0],
[0, 0, 4, 10, 12, 6, 0, 0],
],
request: “evaluate”
}

After the evaluation in the Jetson module, the next function processes the HTTP response and sends information to the desktop application using web-socket and store all data to the database.

#### 2.4.2. Data Provisioning in Neural Network Training

**Figure 13:**
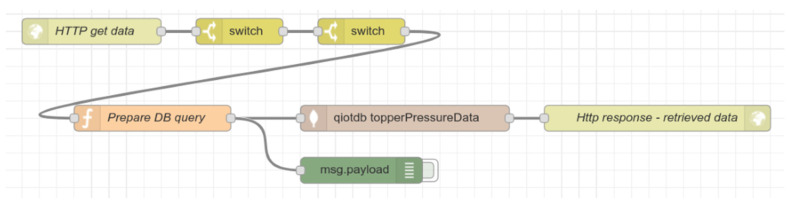
The Node-RED flow providing the application programming interface (API) for neural network based on HTTP request–response communication model.

There is one more flow in our application. This flow serves as an API point for neural network training purposes. The Figure 13 shows this flow.

The flow starts with HTTP in node, where there are two compulsory parameters in the HTTP request, the pose identification, and request type. Based on the request tag, the function block Prepare DB query prepares the query for getting the data from the database. There are two valid request types, training, and testing. For training purposes, 70% of the data are used for one pose. The rest (30%) use the neural network for testing. The HTTP out node response contains all valid data from databases in JSON format.

#### 2.4.3. Desktop Application

**Figure 14:**
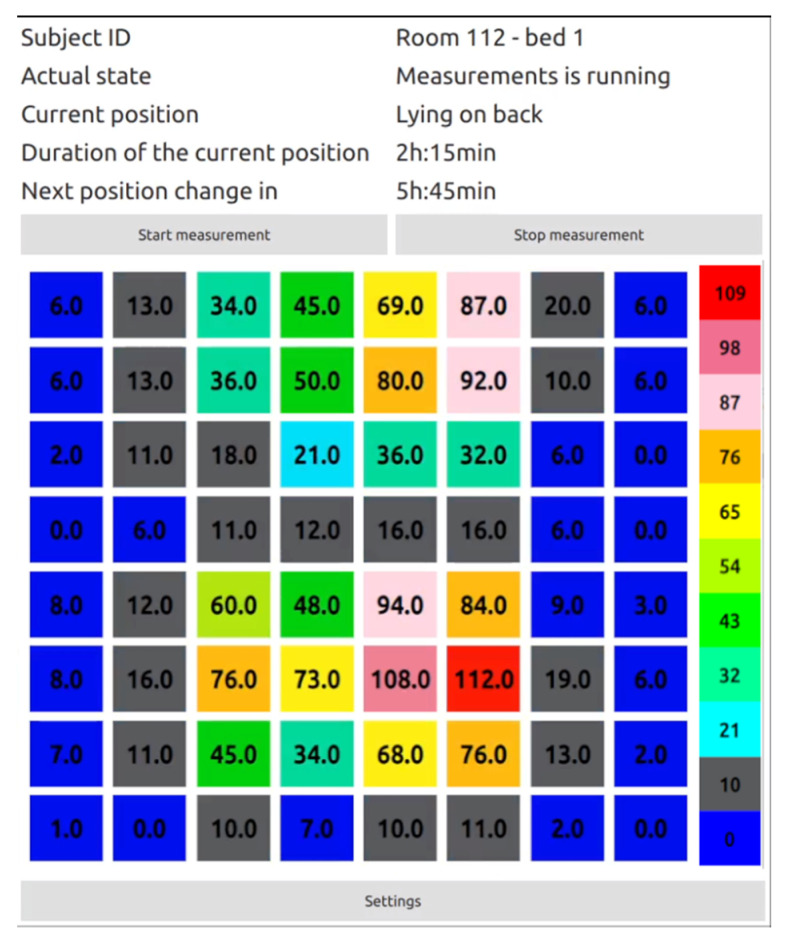
Desktop application for informing the staff about the subject identification, current state, current position, duration of the current position, and time to the next position change.

We created a desktop application for the operating staff using the Qt framework. Figure 14 illustrates a primary application screen.

The application is using HTTP request–response model to send commands to start or stop measurements. Actual data like subject identification, current state, current position, duration of the current position, and time to the next position change give the staff the information about the subject lying on the smart topper. There is also a graphical representation of the current state of the lying subject. The red color represents the maximal pressure, while the blue color represents minimal or non-pressure on a particular sensor in the matrix of sensors. The displayed value represents the pressure in numerical form. This number goes from 0 to 140. The 0 value describes the state when there is no pressure applied to the sensor. The value of 140 represents the maximum load, which the sensor is capable of measuring.

## 3. Experimental Results

We will present the obtained experimental results in this section. First, we will provide the results from the single sensor resistive response measurements. Then, we will describe the data acquisition for training purposes. In the end, we will provide the results for lying posture detection using a Convolutional Neural Network (CNN) [21,22].

### 3.1. Single Sensor Resistive Response Measurements

**Figure 15:**
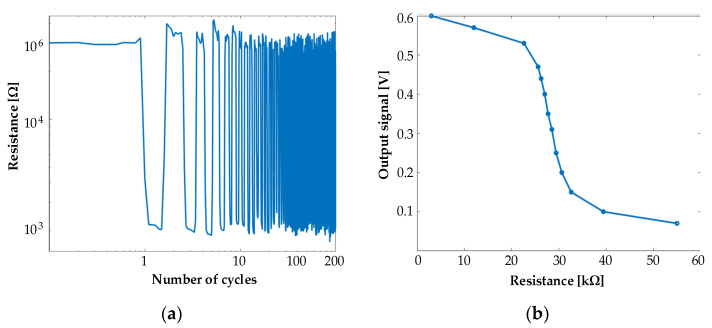
(**a**) The resistive response of a single-sensor element to a load of 10 N over 200 cycles. (**b**) The value of output signal as a function of the changing resistance.

We performed the resistive response of a single-sensor element to a load of 10 N over 200 cycles. We measured the sensor resistance every 100 ms. Figure 15a illustrates the obtained response. There is a little variability in zero load resistance and also with the applied load. The minimum measured resistance during the response measurements was 1.5 kΩ, but with more applied force, the resistance goes down to 300 Ω, and this value is stable. The zero-load variability makes no difference in our system. The proposed hardware solution is most sensitive in the range of 10 kΩ, more precisely from 22 to 32 kΩ. Therefore, small variation in a range more than 32 kΩ does not affect the system. This was the reason why we added a 22 kΩ resistor in the front of trans-impedance amplifiers.

We determined the value of the resistor based on the performed analysis of the output value change in the response of changing the resistance with a potentiometer. Figure 15b describes this observation.

### 3.2. Data Acquisition

**Figure 16:**
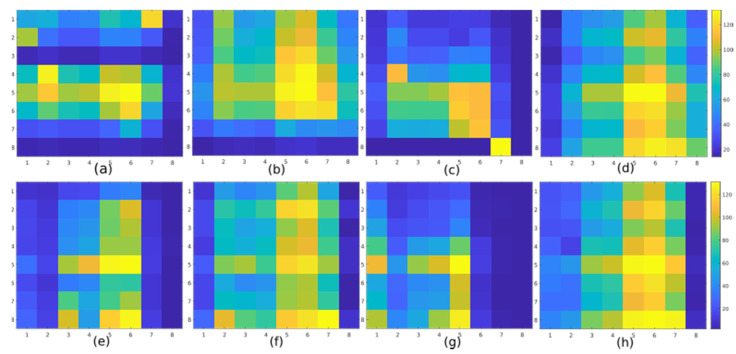
Pressure distribution example for lying postures, **Supine:** (**a**) subject 1, (**b**) subject 2, **Left side:** (**c**) subject 1, (**d**) subject 2, **Prone:** (**e**) subject 1, (**f**) subject 2, **Right side:** (**g**) subject 1, (**h**) subject 2.

We had to create a dataset based on our proposed smart topper for neural network learning. Together, we collected data from 21 subjects, of which 3 were women and 18 men. The subjects’ weights varied from 45 to 125 Kg. The database was divided into training and a test set randomly. In our case, we used data collected from 15 subjects for training. The data collected from the remaining six subjects serve to test the performance of the trained model. In our work, we determined four different postures for laying person classification, namely supine, the left side, prone, and the right side. Figure 16 shows samples of pressure images acquired from different subjects lying in one of the four postures. As mentioned above, the subject’s weight varies a lot. Based on the wide weight variance, posture classification is a very challenging task. For example, when we compared the pressure distribution of one posture for the subject with the lowest weight and the heaviest subject, the difference was enormous.

We created an automated graphical tool for data collection and annotated storage in the MongoDB on the server and used the hardware described in the previous section on hardware design. The measurements repeat every second. The measuring range is from zero to 140. Zero means minimal or no pressure applied on the sensor, while 140 represents the maximal pressure on the sensors. Each subject spent 30 s in each posture. After ten seconds of measurement, we asked the subject to stand up and lay down in the same posture but in a different position. The subject could lay in any desired position for the given posture that feels comfortable. In the end, we had 120 samples for each subject, 30 samples per posture. For training purposes, 450 samples were used for one pose. The remaining 180 samples use neural network for testing (see Table 3).

### 3.3. Proposed Methodology Using Modified CNN

**Figure 17:**
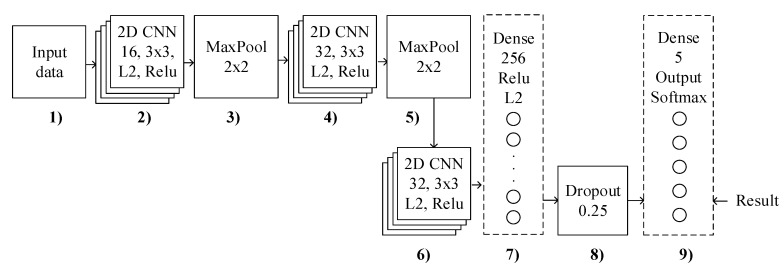
The block diagram of the modified convolutional neural network (CNN).

This section describes the structure of the proposed CNN (Figure 17). The convolutional neural network classifies an input image into categories: supine, the left side, prone, and the right side. We performed the experiments using modified CNN and the precision, recall, and F1 parameter represents the achieved results. The model of the modified CNN consists of these primary layers:

keras.Input(shape=input_shape),
layers.Conv2D(16, kernel_size=(3, 3), activation=“relu”),
layers.Conv2D(32, kernel_size=(3, 3), activation=“relu”),
layers.Conv2D(32, kernel_size=(3, 3), activation=“relu”),
layers.Flatten(),
layers.Dropout(0.25),
layers.Dense(num_classes, activation=“softmax”),

These basic operations (layers) form the core of almost every convolutional neural network. The task of the first layers is to extract the necessary samples from the input data. These first three layers are usually repeated several times. As you can see in Table 4, the layer consists of several functional maps. The main task of the functional map is the extraction of selected features using a convolution filter. The samples are then combined into feature map within the fully interconnected layers and perform a final operation called classification [23,24,25]. This modified CNN divides into nine main blocks (see Figure 17):

1. Reshaping the input data as vectors.
2. This block describes the 2D CNN layer which has 16 feature maps with a 3 × 3 kernel dimension. This layer creates a feature map to predict the class probabilities for each feature by applying a filter (kernel). The activation function uses the Rectified Linear Unit.
3. The MaxPooling layer with a size of 2 × 2 was used. These layers are inserted between the individual convolution layers to reduce the computation time.
4. This block uses the 2D CNN with the same parameters as in step 2, but the number of feature maps into value 32 was doubled.
5. The MaxPooling layer with a size of 2 × 2 was used. These layers are inserted between the individual convolution layers to reduce the computation time. The output from the feature map of the last convolution layer or subsampling layer (MaxPooling layer) is transformed into a one-dimensional vector.
6. In the block 6, the 2D CNN with same parameters as in step 4 was used.
7. In this step, dense layer with non-linearity activation function (ReLU) was used.
8. In this step, the dropout layer with a probability of 0.25 was added to prevent overtraining.
9. The last layer is the SoftMax function. The goal of this layer is to normalize the output of individual neurons to match the obtained probabilities (validation of the training progress).

In the other words, our modified CNN is trying to learn more and more abstract features. In the initial layers this network encodes low-level features such as edge detectors. In the following layers, the features for shapes such as multicolor gradients are described. In the last layers, there are features for individual objects or very complex shapes. Firstly, the input for CNN is data (8 × 8) which passes through the convolutional layer. These convolutional layers consist of a set of filters that are used to extract local image features. The activation function in the neural network determines the values of the outputs of the individual neurons based on their internal potential (the internal potential is calculated by multiplying the weights with the input). In our case, the Rectified Linear Unit (ReLu) as an activation function was used (this is a nonlinear function). Next, CNN applies the Max Pooling layer (sub-sampling) layer. In the Pooling operation, the feature map is divided into several sub-windows. Only the maximum value of each window is then left. The outputs from the individual windows are combined to create a new scaled-down feature map. When we apply this to all maps, a new set of feature maps is created, which then forms as an input to the next convolution layer, where the whole process is repeated. After the Max Pooling operation, the convolutional layer output is flattened through a fully connected layer. Finally, there is a SoftMax output layer for image classification. The SoftMax function assigns to each class the probability that the input image belongs to the appropriate class. The sum of all probabilities is equal to 1. The output from last layer (fully connected layer) can be used as a descriptor for other machine learning algorithms [24,25,26].

The confusion matrix (Table 5) holds the results from each tested method. This matrix summarizes the result of the classification and has rows indexed by output variable classes (reality) and columns by classes that the model predicted (estimation/prediction). The rows of the confusion matrix represent the actual class (supine, the left side, prone, and the right side). In our case, 180 samples in each row for testing were used. On the other hand, the column of confusion matrix represents the predicted class (supine, the left side, prone, and the right side).

Based on the experiments carried out, the supine posture classification achieved the best results at 0.84 in the term of the F1 parameter (see Table 6). On the other hand, the prone posture classification gets the lowest value at 0.79 from all postures (see Table 5). The resulting accuracy is obtained as the sum of correctly predicted samples to the sums of all samples (0.82).

**Figure 18:**
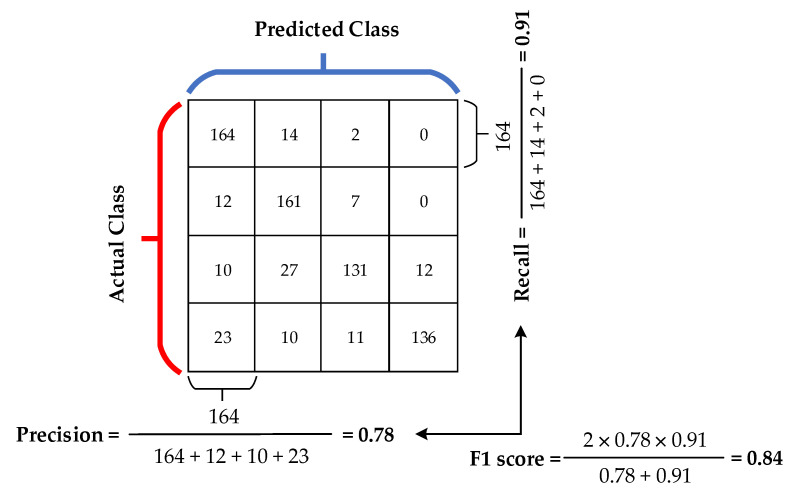
The evaluation metrices (precision, recall, F1 score).

The parameters (P, R, F1) were calculated from the obtained values (see Table 6). The precision is the ratio between True Positive (TP) and the sum of positive data (True Positive (TP) + False Positive (FP)). The recall is the ratio between True Positive (TP) and the sum of data from the actual class (True Positive (TP) + False Negative (FN)) as shown in Figure 18. The F1 score is a combination of precision and recall (weighted average value of precision and recall). The TP value indicates the number of correctly classified patterns of the class true, the value FP indicates the number of incorrectly classified patterns of the class true. The value TN indicates the number of incorrectly classified patterns of class false, and the value FN represents the number of incorrectly classified patterns of class false.

## 4. Discussion

We have presented a smart IoT system for detecting the position of a lying person using novel textile pressure sensors. We introduced our novel textile pressure sensor based on the electrically conductive yarn and the Velostat. Our sensors showed a relatively stable resistive response. When we compare it with the pressure sensor presented in [7], which is also fabricated by sewing, their sensor provides a more stable resistive response to ours and is easier to manufacture. On the other hand, our sensor design is more robust and tear-resistant. Other works [10,11,12,15] use Velostat as the sensing element in their pressure sensors, but they do not use the sewing machine. In the comparison with other presented sensors, our sensor showed the biggest difference in measuring the resistance with the no-load and maximal load. For example, the resistance changes from 6 kΩ to approximately 700 Ω for the sensor presented in [10], respectively, from 2.5 kΩ to 400 Ω for the sensor proposed in [11]. The advantage of our sensor is also that it is based on materials which are gentle on the human skin and can be in direct contact with it.

To obtain the pressure distribution of the lying subject, we used sixty-four sensors. We created a matrix of sensors with eight rows and fabricated it on the base fabric. The final topper consisted of 40 mm thin polyurethane foam for a standard mattress of 2000 by 900 mm where the matrix of sensors is on the top side. Authors in [1] rely on the commercially available mattress for getting the laying person pressure distribution. The mattress has significantly more sensing points over our solution (1728). On the other hand, the works presented in [2,3,4] uses their version of the bedsheet sensing element and the number of the sensing elements used is 45 [2], 12 [3], 16 [4], respectively. They used commercially available sensors from the FSR family and only attach these sensors to the bed or sheet. The work presented in [1,2,3,4], and also our solution, use a non-invasive method and without electronics attached to the subject for the prevention of bedsores. In comparison, the works presented in [5,6] are also non-invasive, but they use an electronic component that needs to be attached to the subject. This can cause some level of discomfort for long-term patients.

In the end, we presented a complex solution for bedsores prevention based on our novel pressure sensor and deep learning. We determined four different postures for laying subject classification, namely supine, the left side, prone, and the right side. We created a challenging dataset and collect the data from 21 subjects. Three of them were women, and 18 were men. The dataset is challenging because the subject weight varies from 45 to 125 kg and the subject could choose the comfortable lying position in each posture. In terms of classification accuracy comparison, the work presented in [1], is the closest to our solution. These authors also used four different postures for classification, and their dataset consists of data from the observation of 12 healthy adults. The big disadvantage shown by classification accuracy comparison is that they have a more precise pressure distribution over our solution. They achieved a high testing prediction accuracy of 97.9% while our solution’s accuracy is 82.22% with the best F1 score of 84%. On the other hand, the solution presented in [2] achieved the lying posture recognition 87.3% and they have only three different postures, left lateral, supine, and right lateral. The solution based on a deep learning approach and an electronic component attached to the subject [5] achieved a high accuracy of 99.56% with F1 1.00%. They used six different postures for classification, where they added sitting and movement posture over our solution. The accelerometer-based solution presented in [6] also achieved a high F1 score that ranges from 95.2% to 97.8%. Overall, the accelerometer-based solution gets better classification results over the sheet-based solution, but there is a disadvantage that the subject has to wear a piece of electronic equipment.

## 5. Conclusions

In this paper, we presented a complex IoT-based solution for detecting the position of a lying person. We produced a smart topper based on our novel pressure sensor for measuring the pressure distribution of the lying person. The novel sensor is based on the electrically conductive yarn and the Velostat. The performed experiments indicate a stable resistive response. We demonstrated the functionality of the whole solution and presented the application to the operating staff using the challenging dataset, which consists of data from 21 subjects. The modified CNN network classifies the collected data into one of the four lying postures. Our modified CNN achieved an overall accuracy of 82.22% and with the best F1 score of 84%.

## Figures and Tables

**Table 1:** The definition of the sensor’s abbreviations.

Abbreviation	Definition
P	Physical quantity, P—pressure
R	Measuring principle, R—resistance, C—capacitance
4	Generation, 1,2,3,4
B	Version, A,B … L

**Table 2:** Description of sensing node design based on the resistivity.

Mark	Description	Layers	Physical Quantity	Fixation	Uncompressed	Compressed
PC2D	*ϕ* 40 mm, stitch pattern: line	2	Capacity mode	Glue	5.5 pF	10.0 pF
			Resistivity mode with Velostat		6.5 kΩ	5.0 kΩ
PC3H	*ϕ* 40 mm, stitch pattern: kite	3	Resistivity mode with Velostat	Glue	7.5 kΩ	1.2 kΩ
PC3I	*ϕ* 40 mm, stitch pattern: kite soft ironing	3	Resistivity mode with Velostat	Glue	8.5 kΩ	4.3 kΩ
PC3J	*ϕ* 40 mm, stitch pattern: kite hard ironing	3	Resistivity mode with Velostat	Glue	105 kΩ	1.0 kΩ
PC3K	*ϕ* 30 mm, stitch pattern: kite strong fixation	3	Resistivity mode with Velostat	Glue	1.6 kΩ	7.0 Ω
PR4A	*ϕ* 30 mm, stitch pattern: line soft fixation	3	Resistivity mode with Velostat	Stitch	22.0 kΩ	200.0 Ω
PR4B	*ϕ* 40 mm, stitch pattern: line less fixation	3	Resistivity mode with Velostat	Stitch	400.0 kΩ	4.0 kΩ
PR4C	*ϕ* 40 mm, stitch pattern: line less fixation	3	Resistivity mode with Velostat	Stitch	10.0 MΩ	5.0 kΩ

**Table 3:** Dataset (four different postures).

Posture Type	Class	Train	Test
Supine	1	450	180
Left side	2	450	180
Prone	3	450	180
Right side	4	450	180

**Table 4:** The structure of the modified CNN (layers description).

Layer Type	Output Shape	Parameters
conv2d_21 (Conv2D)	(None, 6, 6, 16)	160
conv2d_22 (Conv2D)	(None, 4, 4, 32)	4640
conv2d_23 (Conv2D)	(None, 2, 2, 32)	9248
flatten_7 (Flatten)	(None, 128)	0
dropout_7 (Dropout)	(None, 128)	0
dense_7 (Dense)	(None, 4)	516

**Table 5:** The achieved results using modified CNN for data classification (confusion matrix).

Class (Actual/Predicted)	Supine	Left Side	PRONE	Right Side
Supine	**164**	14	2	0
Left side	12	**161**	7	0
Prone	10	27	**131**	12
Right side	23	10	11	**136**

**Table 6:** The evaluation criterion of the modified CNN.

Class	Precision	Recall	F1 score
Supine	0.78	0.91	0.84
Left side	0.76	0.89	0.82
Prone	0.87	0.73	0.79
Right side	0.92	0.76	0.83

## Data Availability

The data presented in this study are available on request from the corresponding author.
